# Supplementary material for: Empowering medical students: Peer-Led OSCE reduces anxiety and may enhance test performance
Source: PLoS One. 2026 Feb 12;21(2):e0340407. doi: 10.1371/journal.pone.0340407 (PMC12900361; doi:10.1371/journal.pone.0340407)
Supplement: Attachment — Submitted filename: Reviewer_Comments_PONE-D-25-10796.pdf [file pone.0340407.s002.pdf]

Reviewer Comments on the Manuscript
"Empowering Medical Students: Peer-Led OSCE Reduces Anxiety and Enhances Test Performance" (PONE-D-25-10796)

General Assessment
This is a well-conceived and relevant study addressing the impact of peer-led mock OSCEs on anxiety and test performance among medical students. The topic is timely and contributes to the literature on cost-effective and student-centered assessment preparation strategies. The manuscript is clearly structured and presents promising results. However, several points should be addressed to strengthen the scientific rigor, clarity, and interpretability of the findings.

Major Points

Study Design and Potential Bias
The non-blinded design and the voluntary nature of the intervention group raise concerns about possible selection bias. Although this limitation is acknowledged, it deserves deeper consideration.
Recommendation: Provide more detail on how baseline characteristics were compared between groups and whether any pre-intervention anxiety or performance indicators were available. Clarify how the study design attempted to minimize self-selection effects.

Generalizability
The study is based on a single institution with a relatively small and predominantly female sample, which limits generalizability.
Recommendation: Clearly state these limitations in the discussion and suggest future multicenter or larger-scale studies to validate the findings.

Statistical Reporting
While appropriate statistical tests were used, key measures such as effect sizes and confidence intervals are missing. These are essential for interpreting the magnitude and clinical relevance of the effects.
Recommendation: Include effect sizes (e.g., Cohen's d) and 95% confidence intervals for primary and secondary outcomes.

Qualitative Insights Missing
The intervention's success likely depends on psychological and educational mechanisms that are not captured by quantitative data alone.
Recommendation: If any qualitative data (feedback from students, open-ended responses) were collected, consider including a brief summary. Otherwise, recommend this as a direction for future research.

Subgroup Analysis within Intervention Group

The manuscript includes both “examiners” and “examinees” within the intervention group but does not thoroughly compare outcomes between these roles. Recommendation: Expand on this point in the results and/or discussion, even if the study was underpowered to detect significant differences.

Minor Points

Language and Style

Minor grammatical errors and awkward phrases appear throughout the text. Recommendation: A thorough English language review is advised to improve flow and clarity.

Title Wording

The current title implies causation, which may not be fully supported given the quasi-experimental nature of the study. Recommendation: Consider revising to “Peer-Led OSCE May Reduce Anxiety and Improve Test Performance.”

Terminology Consistency

The terms “mock OSCE” and “formative OSCE” are used interchangeably. Recommendation: Choose one term and apply it consistently to avoid confusion.

Figure Legends and Tables

Some figure legends (e.g., for anxiety and performance scores) lack sufficient detail to be understood independently. Recommendation: Expand legends to define all abbreviations and describe what is shown.

Conclusion and Recommendation

The manuscript presents a valuable and innovative approach to medical education and assessment preparation. With minor revisions to address statistical reporting, editorial clarity, and a deeper analysis of potential biases and subgroup roles, it will be suitable for publication.

Recommendation: Accepted with minor changes.
